# Higher risk sexual behaviour is associated with unawareness of HIV-positivity and lack of viral suppression – implications for Treatment as Prevention

**DOI:** 10.1038/s41598-017-16382-6

**Published:** 2017-11-23

**Authors:** Helena Huerga, Emilie Venables, Jihane Ben-Farhat, Gilles van Cutsem, Tom Ellman, Chris Kenyon

**Affiliations:** 1 Clinical Research Department, Epicentre, Paris, France; 2 Southern African Medical Unit, Médecins Sans Frontières, Sans Frontières, South Africa; 3 Division of Social and Behavioural Sciences, School of Public Health and Family Medicine, University of Cape Town, Cape Town, South Africa; 4 Centre for Infectious Disease Epidemiology and Research, University of Cape Town, Cape Town, South Africa; 5 HIV/STI Unit, Institute of Tropical Medicine, Antwerp, Belgium; 6 Department of Medicine, University of Cape Town, Cape Town, South Africa

## Abstract

Efficacy of Treatment as Prevention Strategy depends on a variety of factors including individuals’ likelihood to test and initiate treatment, viral load and sexual behaviour. We tested the hypothesis that people with higher risk sexual behaviour are less likely to know their HIV-positive status and be virologically suppressed. A cross-sectional population-based survey of individuals aged 15–59 years old was conducted in 2013 in KwaZulu-Natal, South Africa. A two-stage cluster probability sampling was used. After adjustment for age and sex, lack of awareness of HIV-positivity was strongly associated with having more than one sexual partner in the preceding year (aOR: 2.1, 95%CI: 1.5–3.1). Inconsistent condom use was more common in individuals with more than one sexual partner (aOR: 16.6, 95%CI: 7.6–36.7) and those unaware (aOR: 3.7, 95%CI: 2.6–5.4). Among people aware of their HIV-positivity, higher risk sexual behaviour was associated with lack of viral suppression (aOR: 2.2, 95%CI: 1.1–4.5). Risky sexual behaviour seems associated with factors linked to poor health-seeking behaviour which may have negative implications for HIV testing and Treatment as Prevention. Innovative strategies, driven by improved epidemiological and anthropological understanding, are needed to enable comprehensive approaches to HIV prevention.

## Introduction

The province of KwaZulu Natal (KZN) in South Africa has one of the highest HIV prevalence rates in the world: 27.9% in 15–49 year olds in in 2012^1^. HIV incidence in KZN remains high (2.6 new infections per 100 person-year for the period 2004 to 2011)^2^. In recent years HIV prevention efforts in the country have placed increasing emphasis on interventions such as increasing the proportion of the population who know their HIV status (Serostatus Approach to Fighting the Epidemic - SAFE) and increasing the antiretroviral (ART) coverage in the form of TasP (Treatment as Prevention). South Africa’s HIV testing programme - despite its challenges - has made considerable progress with an estimated 9.5 million tests being performed annually^3^, 76% of HIV-positive persons being aware of their diagnosis and the country being on target to increase this percent to 90% by 2020^4^. TasP has been shown to dramatically reduce the risk of HIV transmission at the level of couples^5^. At population level, studies have also demonstrated that TasP can reduce HIV incidence^2^. A number of modelling studies have gone further and suggested that upscaling TasP would lead to the elimination of HIV transmission in South Africa^6,7^.

A relatively underexplored aspect of these models is their assumption that the sexual and treatment behaviour of individuals who take up ART in TasP does not differ from those who do not. If the individuals who engage in the highest risk behaviour are less likely to be aware of their HIV positive status and less likely to be virologically suppressed if they are aware, then this will limit the efficacy of TasP in controlling HIV transmission. The same logic could apply to the efficacy of SAFE^8^. These considerations may help explain the recently reported negative interim results of a large cluster randomized controlled trial of universal TaSP in KZN^9,10^.

In this paper we test the assumption that people with higher sexual risk behaviour are less likely to know their HIV-positive status and be virologically suppressed.

## Methods

### Design and population

This is a secondary analysis that uses data from a cross-sectional population-based study conducted in the King Cetshwayo District, KZN, between July and October 2013^11^. People aged 15–59 years old living in Mbongolwane and Eshowe Health Service Areas, uMlalazi Municipality in the province of KwaZulu Natal, were eligible for participation. Mbongolwane is a rural area of approximately 120,000 inhabitants and the main town in the municipality is Eshowe, located about 140 km north of Durban. According to the 2011 Census^12^, 61,179 people aged 15 to 59 were living in 25,106 households in the area covered by the survey.

A two-stage stratified cluster sampling was used^13^. A total of 125 clusters of 25 households each were included. The number of clusters per administrative area was selected with probability proportional to population according to the 2011 Census. Google Earth maps from 2011 were used to sample the households to be visited. Field staff used Global Positioning System (GPS) receivers to find the geographical coordinates of each household.

### Study procedures

**Figure 1:**
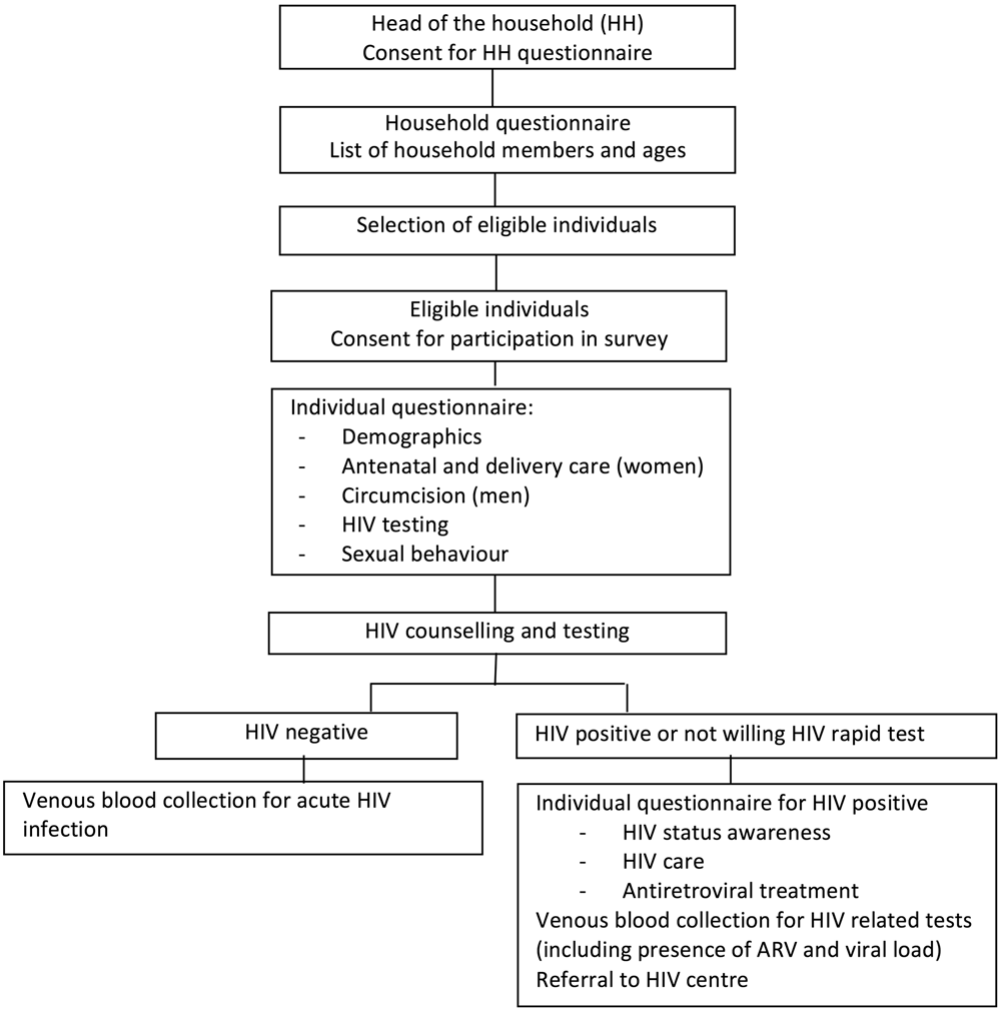
Flow chart for survey procedures.

Prior to the start of the survey, community engagement activities were organised to inform the community and a five-day training course was provided to the field interviewers and nurses. As a first step, after providing consent the head of the household was asked to list all household members and provide their age so that the eligible individuals for the survey could be identified (Fig. 1). All eligible individuals were asked to give individual consent and those agreeing to participate in the survey were included. Participants were asked questions about their demographics, antenatal and delivery care (only women), circumcision (only men), previous HIV tests, and sexual behaviour through face-to-face structured questionnaires. Interviews were conducted at home in a private place chosen by the participants. Sexual behaviour questions were asked at the end of the general interview and before the questions related to the HIV status and treatment. The questions included: when was the last sexual intercourse; condom use, type of relationship and month and year of the first and last sexual intercourse with the last 3 sexual partners in the 12 months preceding the interview; and total number of sexual partners in the 12 months preceding the interview. All individuals participating in the survey were tested for HIV even if they already knew their status. HIV rapid tests were conducted at the participants’ house by certified lay counsellors using Determine Rapid HIV-1/2 Antibody as a screening test followed by Unigold Rapid HIV test for confirmation if positive. Counselling was conducted before and after the HIV test and the results were given to the participant. Individuals not willing to have an HIV rapid test or who did not want to know the result could opt for anonymous HIV testing at the laboratory. ELISA was used for anonymous HIV testing and to confirm the positive HIV rapid test results. Participants with a positive result on the rapid HIV test and those who opted for anonymous HIV testing were asked about their HIV awareness and antiretroviral treatment and had full blood specimens collected to perform other HIV related tests including presence of antiretroviral drugs (liquid chromatography tandem mass spectrometry with a limit of quantification of 0.04 micrograms/ml) and viral load (VL - NucliSens EasyQ HIV-1 v2.0 assay from Biomerieux). All viral load results are reported as copies/mL. Participants were informed that the results of these tests would be available at the closest health centre and were given a letter to collect them. Antibody negative participants also had a blood specimen collected and were tested for acute HIV infection using Roche AMPLISCREEN individual Nucleic Acid Amplification Testing (NAAT) testing with the Roche CAP/CTM method on specimens in positive (5 member) pools tested.

### Ethics

Written informed consent was sought prior to the enrolment of participants. The protocol was approved by the University of Cape Town Human Research Ethics Committee (HREC), the Health Research Committee of the Health Research and Knowledge Management Unit of KZN DOH, and the “Comité de Protection de Personnes”, Paris, France. All methods were performed in accordance with the relevant guidelines and regulations.

### Definitions

We considered condom use to be consistent when individuals declared using condoms at the last sexual intercourse with all partners in the preceding year, and inconsistent otherwise. Sexual behaviour in the preceding year was categorized in four risk groups: Group 1 - no sexual intercourse in the preceding year; Group 2 - one sexual partner with consistent use of condoms; Group 3 - one sexual partner with inconsistent use of condoms. Of those with more than one sexual partner only 7 of 165 used condoms consistently. We therefore defined Group 4 (high risk behaviour group) as those with more than one sexual partner regardless of condom usage. Individuals whose HIV viral load was 1000 and above were defined as virally unsuppressed. Individuals were defined as mobile if they had changed their place of residence in the previous 10 years^12^ or if they were visitors to the household. Individuals with ARV present in blood were classified as aware of their HIV status regardless of the reported declaration of awareness.

### Statistical analyses

All statistical analyses were adjusted for clustering. Descriptive analyses are presented here with 95% confidence intervals (CI). Two-sample t test and Pearson chi-square statistics were used to compare continuous and categorical descriptive outcomes as appropriate. We stratified the population in 3 age groups: 15–19 years, 20–34 years and 35–59 years. The intervals of years in the categories are not homogenous because the relation between age and sexual behaviour is not linear. The category 15–19 years represents adolescents, whilst the 20–34 category represents the young adult population and the category 35–59 years the middle aged adult population. Sexual behaviour in South Africa and elsewhere have been shown to vary between these age categories^14–17^. We used multiple logistic regression models to identify factors associated with having more than one sexual partner in the preceding year and being virally unsuppressed. In addition, we used multiple logistic regression models separately in women and men to identify factors associated with using condoms consistently with all sexual partners and to assess the relation between viral suppression and sexual behaviour using the sexual behaviour groups defined previously. We included the following covariates in the univariate models: age (15–19, 20–34, 35–59 years), gender (women, men), marital status (not living in couple, living in couple), living area (rural, urban), mobility (resident, migrant/visitor) and awareness of HIV positivity (aware, unaware). In addition, the number of partners was included in the model evaluating condom use (≤1 partner, >1 partner) and the sexual behaviour groups 1, 2, 3 and 4 were included in the model evaluating the relation between sexual behaviour and viral suppression. Characteristics associated in univariate analyses with p < 0.25 were included in the initial adjusted models. A decreasing step-wise strategy was used to choose the variables to be included in the final model. Age and sex were included in the final model as an a priori decision in addition to variables with p < 0.10.

Data were entered and checked using Epidata version 3.1 and analysed using Stata 13 (Stata Corp., College Station, Texas, USA).

### Data availability

The datasets analysed during the current study are available from the corresponding author on reasonable request.

## Results

### Study population

Survey response rate was 84.5% (5649/6688) overall, 87.8% (3518/4008) among women and 79.5% (2131/2680) among men. In total, 1423 participants were HIV-positive and therefore included in this analysis. Of them, 1085 (76.2%) were women and 338 (23.8%) men. Median age was 34 years (IQR: 27–42), 70 (4.9%) were aged 15–19 years, 677 (47.6%) 20–34 years and 676 (47.5%) were 35 years or older. In total, 345 (24.3%) were married or living together, 1053 (74.0%) had completed at least primary school, 711 (50.0%) were unemployed, 1142 (80.3%) lived in rural areas and 282(19.8%) were mobile.

### Sexual behaviour, HIV-positivity awareness and viral load

Amongst the HIV-positive individuals, 382 (26.9%) reported no sex in the preceding year, 377 (26.5%) reported one sexual partner and consistent condom use, 497 (35.0%) reported one sexual partner with inconsistent condom use, and 165 (11.6%) reported more than one sexual partner (Table 1). Of the 1038 individuals who reported sex in the preceding year, 123 (11.9%) engaged in concurrent partnerships (partnerships overlapping in time) and 39 (3.8%) reported casual or transactional partners. Concerning condom use, 670 (64.6%) reported condom use with the last partner and 384 (37.0%) with all partners in the preceding year. Among people with sexual activity in the preceding year, more men than women reported having had more than one partner, concurrent partnerships and casual or transactional partners: 34.8% vs 8.9% (p < 0.001), 27.5% vs 6.1% (p < 0.001) and 9.2% vs 1.7% (p < 0.001), respectively. Overall, more men than women reported more than one sexual partner in the preceding year: 29.0% vs 6.2% (p < 0.001) and particularly men aged 20–34 years compared to women of the same age group: 40.7 vs 8.8 (p < 0.001). More men than women also reported inconsistent condom usage: 68.4 vs 61.0 (p = 0.028).

**Table 1:** Sexual behaviour, HIV-positivity awareness and viral load by sex and age groups.

	Women				Men			
	15–19 y (N = 58)	20–34 y (N = 537)	35–59 y (N = 490)	All (N = 1085)	15–19 y (N = 12)	20–34 y (N = 140)	35–59 y (N = 186)	All (N = 338)
Sex in preceding year, n (%)	39 (67.2)	458 (85.4)	259 (53.1)	756 (69.9)	4 (33.3)	127 (90.7)	151 (81.2)	282 (83.4)
No. partners in preceding year, mean [std]	1.1 [0.3]	1.1 [0.4]	1.1 [0.2]	1.1 [0.3]	2 [0]	1.9 [1.8]	1.3 [0.8]	1.6 [1.4]
>1 partner^1^, n (%)	5 (12.8)	47 (10.3)	15 (5.8)	67 (8.9)	4 (100)	57 (44.9)	37 (24.5)	98 (34.8)
Casual/transactional partner^1^, n (%)	0 (0.0)	10 (2.2)	3 (1.2)	13 (1.7)	0 (0.0)	16 (12.6)	10 (6.6)	26 (9.2)
Concurrency^1^, n (%)	4 (10.3)	32 (7.0)	10 (3.9)	46 (6.1)	3 (75.0)	46 (36.8)	28 (18.5)	77 (27.5)
Condom use in last SI^1^, n (%)	20 (51.3)	286 (62.6)	180 (69.5)	486 (64.4)	3 (75.0)	70 (55.1)	111 (73.5)	184 (65.3)
Condom use in all SI^1^, n (%)	11 (28.2)	163 (35.6)	121 (46.7)	295 (39.0)	0 (0.0)	25 (19.7)	64 (42.4)	89 (31.6)
Unaware HIV positivity, n (%)	19 (32.8)	165 (31.0)	57 (11.7)	241 (22.3)	5 (41.7)	68 (48.9)	37 (20.0)	110 (32.7)
Viral load ≥ 1000 copies/mL^2^, n (%)	36 (64.3)	260 (49.5)	130 (26.9)	426 (40.0)	9 (75.0)	92 (67.7)	72 (39.3)	173 (52.3)

^1^Among individuals reporting having had a sexual partner in preceding year.

^2^Among individuals with viral load available.

Overall, 351 (24.8%) individuals were unaware of their HIV positivity and 599 (42.9%) had a viral load of 1000 or above. Unawareness and viral load of 1000 and above were more common among men than women: 32.7% vs 22.3% (p < 0.001) and 52.3% vs 40.0% (p < 0.001) respectively. Young people were also more likely to be unaware or virally unsuppressed. Among individuals aged 15–19 years and 20–34 years, 34.3% and 34.7%, were unaware vs 14.0% of those aged 35–59 years (p < 0.001). Among individuals aged 15–19 years and 20–34 years, 66.2% and 53.3%, were virally unsuppressed vs 30.3% of those aged 35–59 years (p < 0.001).

### Relation between HIV status awareness, sexual behaviour and viral load

Unawareness of HIV positivity was strongly associated with having more than one sexual partner in the preceding year and with high viral load after adjustment for age and sex. Among people reporting more than one sexual partner, 45.5% were unaware of their HIV positivity vs 22.1% among those with fewer partners (p < 0.001). As expected, individuals unaware of their status were virally unsuppressed. Therefore, among people with viral load of 1000 and above, 47.6% were unaware vs 5.9% of those with viral load less than 1000 (p < 0.001). In multivariable analyses, individuals aged 20–34 years, men, those reporting mobility and individuals unaware of their HIV-positive status had a higher odds of having more than one sexual partner (Table 2). In a separate multivariable analysis for women and men after adjustment for age, inconsistent condom use in the preceding year was independently associated with having more than one sexual partner (women aOR: 9.6, 95%CI: 3.4–26.9; men aOR: 23.6, 95%CI: 7.1–78.4) and unawareness of being HIV-positive (women aOR: 3.4, 95% CI: 2.2–5.2; men aOR: 4.1, 95%CI: 2.0–8.7). In addition, individuals of the same age and sex unaware of their HIV-positivity (aOR: 11.9, 95% CI: 8.5–16.8) and individuals with more than one sexual partner (aOR: 1.6, 95%CI: 1.1–2.5) had a higher odds of being virally unsuppressed.

**Table 2:** Factors associated with having more than one sexual partner.

	Unadjusted			Adjusted		
	OR	95%CI	p	aOR	95%CI	p
Age						
35–59 years	Ref			Ref		
20–34 years	2.2	1.5–3.1	<0.001	2.2	1.5–3.3	<0.001
15–19 years	1.8	0.8–3.8	0.140	2.0	0.9–4.6	0.109
Sex						
Women	Ref			Ref		
Men	6.2	4.4–8.7	<0.001	6.7	4.7–9.6	<0.001
Marital status						
Not living in couple	Ref			—	—	—
Living in couple	0.9	0.6–1.3	0.456	—	—	—
Area of residence						
Rural	Ref			Ref		
Urban	1.9	1.3–2.8	<0.001	1.6	1.0–2.4	0.030
Mobility						
Resident	Ref			Ref		
Migrant/visitor	2.4	1.7–3.4	<0.001	2.1	1.4–3.1	<0.001
HIV-positivity awareness						
Aware	Ref			Ref		
Unaware	2.9	2.1–4.1	<0.001	2.1	1.4–3.0	<0.001

**Figure 2:**
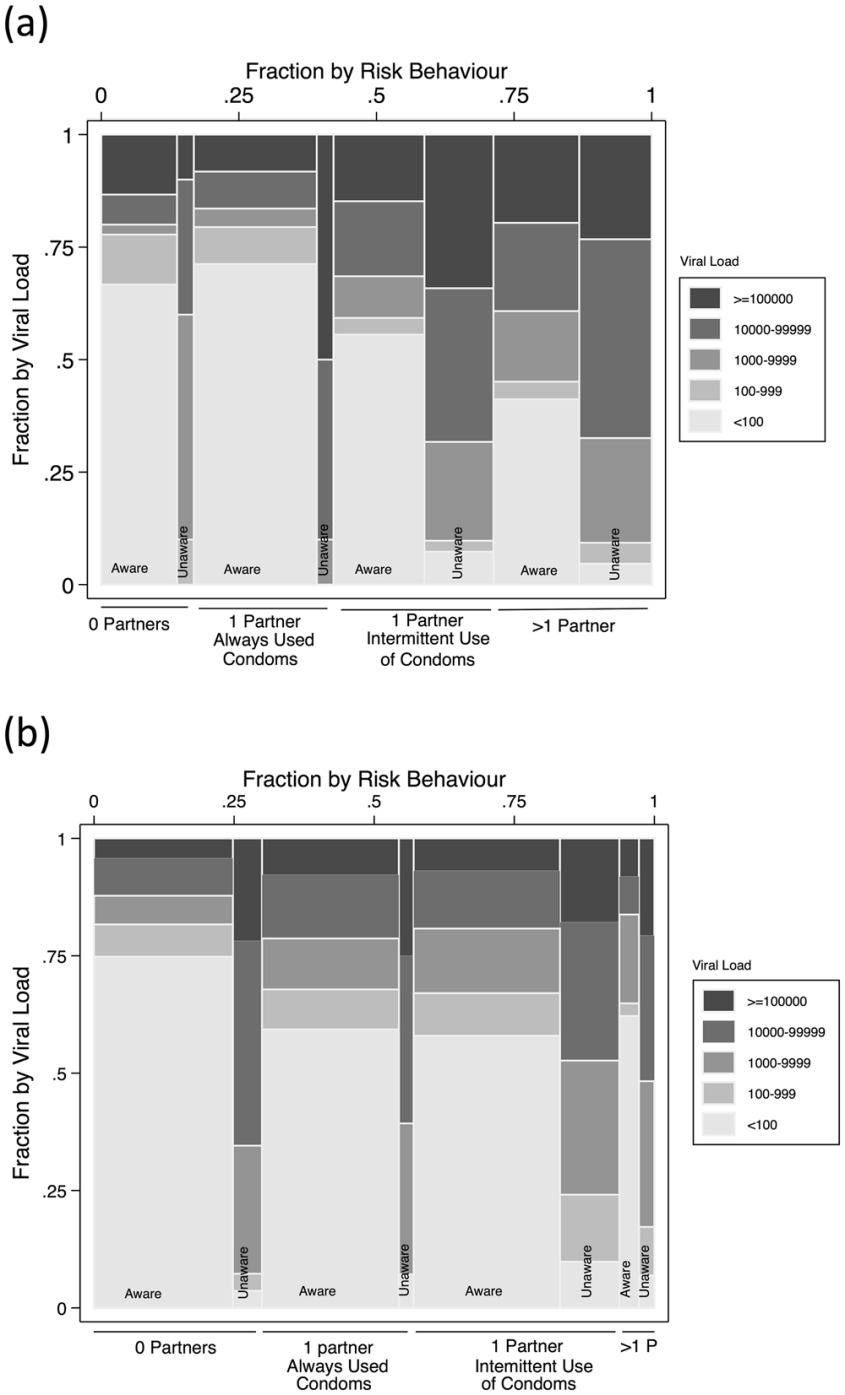
Viral load according to the sexual behaviour and the HIV status awareness in men (**a**) and women (**b**). The width of the columns represents the proportion of individuals aware and unware within each category of sexual behaviour.

In men, the risk of transmitting HIV was linked to high risk sexual behaviour, unawareness of HIV status and high viral load. Overall 29.0% of men had more than one partner (96.9% of them with inconsistent use of condoms) and 52.3%/34.7% a viral load of ≥1000/≥100,000 (Fig. 2a). The percent of men virally unsuppressed increased with increasing risky sexual behaviour, from 34.6% and 31.0% in behaviour groups 1 and 2 (no sexual intercourse in the preceding year and one sexual partner with consistent use of condoms) to 61.5% in group 3 (one sexual partner with inconsistent use of condoms) and 71.9% in group 4 (more than one sexual partner regardless of condom usage), p < 0.001. Since viral load was higher in those unaware of their HIV status (and therefore unable to be on ART), this association was partly determined by an increasing proportion of people who were unaware of their HIV-status (from 19.6/14.1% in groups 1/2 to 43.3/45.9% in groups 3/4; p < 0.001). In a logistic regression model adjusted for age, behaviour groups 3 and 4 had a higher odds of being virally unsuppressed compared to behaviour group 1 (group 3 aOR: 2.7, 95%CI: 1.1–6.4; group 4 aOR: 4.0, 95%CI: 1.7–9.8). When limiting the analysis to those aware of their HIV-status, there was however still an increase in percent of virally unsuppressed from 22.2/20.6% in groups 1/2 to 40.7/55.7% in groups 3/4 (p < 0.001).

In women, the risk of transmitting HIV was mainly linked to high viral load. Only 8.9% of women (vs 29% of men) had more than one partner (94.0% of them with inconsistent use of condoms) and 40.1% had a viral load of 1000 or more (23.7% of them of 100,000 or more) (Fig. 2b). As with men, the percent of virally unsuppressed women increased with increasing risk behaviour (from 31.5/38.1% in behaviour groups 1/2 to 45.9/56.1% in groups 3/4; p < 0.001). This association was partly determined by an increasing proportion that were unaware of their HIV-status (from 18.8/10.7% in groups 1/2 to 29.8/44.8%in groups 3/4; p < 0.001). In a logistic regression model adjusted for age, behaviour group 2 had a higher odds of being virally unsuppressed compared to behaviour group 1 (aOR: 1.5, 95%CI: 1.0–2.3). However behaviours groups 3 and 4 were not statistically associated with viral unsuppression (group 3 aOR: 1.3, 95%CI: 0.9–1.8; group 4 aOR: 1.3, 95%CI: 0.7–2.6). When limiting the analysis to those aware of their HIV-status there was an increase in percent of women virally unsuppressed but unlike men only when risk behaviour group 1 (no sex in previous year) was compared to the other 3 groups (from 18.3% in group 1 to 32.2/33.0/35.1% in groups 2/3/4; p < 0.001). Only 3.5% of women reported more than one partner and had a viral load of 1000 or more.

In multivariable analysis in people aware of their HIV-positive status, after adjusting for ARV drug presence in blood, age and sex, individuals with more than one sexual partner had a two times increased risk of being virally unsuppressed (aOR: 2.2, 95%CI: 1.1–4.5).

## Discussion

In keeping with previous studies, we found that those unaware of their HIV-positivity were more likely to report higher risk behaviour than those aware^18,19^. A novel finding of this study was that higher risk behaviour was also associated with higher viral loads in those who were aware of their HIV-positivity. Whilst the association between unawareness and higher risk may be partly or fully explained by risk reduction in those who are aware of their positive status, this cannot explain the association when the analysis is limited to the aware group^19^. This association is more likely explained by unmeasured factors, whether external or individual, that influence both seeking/taking treatment and risk behaviour. While our study is unable to elucidate the causal pathways, the findings are significant as they reveal potential real-world limitations to both SAFE and TasP.

The findings are particularly salient in the setting of having taken place after a number of sero-awareness campaigns that resulted in 75.2% of the HIV-positive population reporting knowing their status^4,11^. If a ‘core-group’ of individuals with higher risk behaviour is less likely to get tested for HIV, and if found to be HIV positive then be less likely to effectively use ART, then this will make this group more resistant to the impact of SAFE and TasP. This effect should then be included in models that assess the effectiveness of these strategies. The recently reported interim results of ANRS 12249, a community cluster randomized controlled trial of universal TaSP reported no difference in HIV incidence between intervention and control arms^9^. One reason for this was the low linkage-to-care-rate of those newly testing HIV positive (36%). It may be helpful if future analyses of these trials also assess if there is clustering of risk factors such as accepting HIV testing, linkage to care and risk behaviour as we found in our analysis. Alternative strategies may need to be developed to deal with this group.

We found that men and people 20–34 years were more likely to report higher risk behaviours than other individuals. Consistent with other studies, men, particularly those aged 20–34 years, were more likely to report having had more than one partner in the preceding year, concurrency and inconsistent condom use than women^20^. Men and people under 35 years were also more often unaware of their HIV positivity and had higher viral loads. Similar findings have been described in other African settings^21–24^.

One limitation of this study is that sexual behaviour data was collected via face-to-face interviews which have been shown to be susceptible to social desirability bias, which could also be different in women and men and at different ages. It is also possible that a recall bias affected participants’ answers to questions about their sexual behaviour and partners several months previously. There may have also been misunderstandings about the different categories of partner, with under-reporting for transactional/casual sex and over-reporting of these partners as ‘boyfriends’ or ‘girlfriends’. However, sexual behaviour results were consistent with findings of other studies and underreporting of high risk sexual behaviour among women or among individuals at older ages may not change the associations between sexual behaviour, HIV-positivity unawareness and viral unsuppression. Participation in the survey was high, although lower among men. If people with high risk sexual behaviour and low viral suppression were less likely to participate in the survey, the associations found in these analyses and the implications for TasP may be even greater.

## Conclusion

In KZN, higher risk sexual behaviour was associated with HIV-positive status unawareness and among those aware with lack of viral suppression. At the time of the survey the group with the highest potential to transmit HIV were people not on ART, either unaware or aware. High risk sexual behaviour seems associated with other factors linked to poor health-seeking behaviour which may have negative implications for HIV testing and TasP. This effect should be included in models that assess the effectiveness of these strategies. There is a clear need to target HIV prevention interventions, including HIV testing campaigns and treatment programmes, to different groups of people based on the risk factors associated with their sexual behaviour, as well as explore alternative retention and adherence strategies. However, such relationships are unlikely to be easily shifted by a purely health sector driven approach and innovative strategies, driven by epidemiological and anthropological evidence, need to be developed to enable more comprehensive approaches to HIV prevention.
